# Experience of hospitalization of the family with children and adolescents in psychological distress

**DOI:** 10.1590/1980-220X-REEUSP-2022-0457en

**Published:** 2023-10-30

**Authors:** Gabriela Morilhas Barbosa, Aldair Weber, Ana Paula Rigon Francischetti Garcia, Vanessa Pellegrino Toledo

**Affiliations:** 1 Universidade Estadual de Campinas, Faculdade de Enfermagem, Campinas, SP, Brazil.

**Keywords:** Family, Child, Adolescent, Mental Disorders, Empathy, Familia, Niño, Adolescente, Trastornos Mentales, Empatía, Família, Criança, Adolescente, Transtornos Mentais, Empatia

## Abstract

**Objective:**

To learn about the experience of families of children and adolescents in psychological distress facing hospitalization in a psychiatric inpatient unit of a general university hospital.

**Method:**

This is a qualitative phenomenological-social study, with data collection carried out from February to March 2022 through open interviews, analyzed using Alfred Schutz’s framework with the construction of categories of meaning.

**Results:**

Based on the analysis of eight interviews, it was possible to understand changes in the families’ way of life, impacts on their routine, work, and social relationships. Their expectations were about the recovery of mental health and autonomy.

**Conclusion:**

This study allowed us to understand the experience of families faced with the hospitalization of children and adolescents in psychological distress and understand their members’ needs. The need for implementation of care spaces that incorporate the relationship between the multidisciplinary team and the family is highlighted.

## RESUMO

**Objetivo:**

Conhecer a experiência da família de crianças e adolescentes em sofrimento psíquico diante da hospitalização em uma unidade de internação psiquiátrica de um hospital geral universitário.

**Método:**

Estudo qualitativo fenomenológico-social, coleta de dados realizada nos meses de fevereiro a março de 2022 por meio de entrevistas abertas, analisadas sob referencial de Alfred Schutz com construção de categorias de significado.

**Resultados:**

Com base na análise de oito entrevistas, foi possível compreender mudanças no modo de vida das famílias, impactos na sua rotina, trabalho e relações sociais. Suas expectativas versaram sobre a recuperação da saúde mental e autonomia.

**Conclusão:**

Este estudo permitiu conhecer a experiência das famílias diante da hospitalização de crianças e adolescentes em sofrimento psíquico e compreender as necessidades de seus membros. Destaca-se a necessidade da implementação de espaços de cuidados que incorporem a relação entre a equipe multiprofissional e a família.

## RESUMEN

**Objetivo:**

Conocer la experiencia de familias de niños y adolescentes en sufrimiento psicológico ante la internación en una unidad de internación psiquiátrica de un hospital general universitario.

**Método:**

Estudio cualitativo fenomenológico-social, recolección de datos realizada de febrero a marzo de 2022 a través de entrevistas abiertas, analizadas utilizando el referencial de Alfred Schutz con la construcción de categorías de significado.

**Resultados:**

A partir del análisis de ocho entrevistas, fue posible comprender cambios en el modo de vida de las familias, impactos en su rutina, trabajo y relaciones sociales. Sus expectativas estaban puestas en la recuperación de la salud mental y la autonomía.

**Conclusión:**

Este estudio permitió comprender la experiencia de las familias ante la hospitalización de niños y adolescentes en sufrimiento psicológico y comprender las necesidades de sus miembros. Se destaca la necesidad de implementar espacios de atención que incorporen la relación entre el equipo multidisciplinario y la familia.

## INTRODUCTION

Given the clinical complexity of children and adolescents in psychological distress, and based on the understanding that family care practices must prioritize social relationships, it is important that health care professionals articulate their therapeutic plans to the family nucleus^(1)^. In this scenario, those responsible for providing care may experience an overload arising from the mental health diagnosis, which comprises the emotional, economic, and practical aspects to which caregivers are exposed^(2)^. Such overload is related to the need for constant attention and supervision of the daily activities of children and adolescents, who may also be aggressive or present difficult behaviors. In addition, family members have to deal with the stigmatization of madness, giving up their own life^(2)^.

In this context, children and young people who need mental health care seek the services of the Psychosocial Care Network (*RAPS*), which indicate that hospitalization should only be carried out in cases where the possibilities of care in basic community territorial services are exhausted and when the user’s clinical need for access to hospital technology is proven^(3)^. However, with the advent of the COVID-19 pandemic, a study exploring the impacts of this event on adolescents who already had psychological distress showed that some symptoms were exacerbated, such as increased anxiety, sleep difficulties, and panic attacks^(4)^. Furthermore, in addition to the aforementioned event, the vulnerability of *RAPS* is evident in terms of its ability to resolve child and adolescent cases and the changes in social behavior that, enhanced by the pandemic, have led to a reduction in access to mental health services, with the hospital highlighted as one of the resources used^(3,4)^.

When hospitalizing children and adolescents, parents or guardians can remain with their child full time, helping to face a series of challenges that may arise due to hospitalization^(5)^. These challenges are characterized by the exacerbation of unpleasant feelings, such as fear, anguish and anxiety, as well as unfamiliarity with the hospital environment^(5)^. This way, family members have the important role of providing emotional support to children and adolescents, to help them tolerate and face the experience of living a unique moment^(5)^.

However, as a result of the hospitalization process, issues arise, experienced by the family in need for effective adjustments and quick responses, such as changes in routine, the impacts suffered in the emotional, social, and financial spheres, withdrawal and lack of social support, lack of care for other children, marital conflicts, among others^(6)^. Therefore, in mental health care, it is essential to recognize each family’s uniqueness to promote individualized care that involves not only the patient, but also their collective support base and the professionals of the care team^(7,8)^.

Therefore, to provide qualified assistance, the hospital environment must have prepared workers who develop a Unique Therapeutic Project (*PTS*), articulating the patient and their family with other services^(2)^. Nursing professionals, working with hospitalized children and adolescents in psychological distress, have difficulties describing their role in caring for this population^(9)^. Often, their practices remain subordinated to other professional categories with generic interventions, and to the lack of knowledge and skills inherent to mental health care^(9)^. The rigid organizational structure, emotional overload, and the presence of the family or guardians who also need planned assistance are also evident^(9)^.

When caring for hospitalized children and adolescents in psychological distress, the family experiences new events and challenges during this process, and such moments become part of their knowledge base and form part of the biographical aspects of the family world, permeated by the relationships among the subjects^(6,10)^. Also, at this time of hospitalization, the family needs to be taken care of, being able to actively get involved with the hospitalized child or adolescent, being identified as an important support, or even a factor that triggers scenarios of improvement in the health condition^(6–8)^. It is necessary to focus on the people who make up the family nucleus and present themselves as part of the unique care of hospitalized children and adolescents, getting to know their life experiences aiming at providing the best assistance^(6–8)^.

The family context is considered one of the dimensions of the object of work in child and adolescent mental health practices, the result of the displacement of practices that prevented the social and family coexistence of the “crazy” person, towards the valorization and inclusion of the family and significant others in their mental health care. Thus, with regard to psychosocial care and public mental health policies aimed at children and adolescents, the emotional bonds among the subjects who make up the family and share their care reinforce the importance of thinking about the longitudinality of the inclusion of family members in care practices across all network devices^(3,5,8)^.

Therefore, the study is warranted due to the need to include families in the care of children and adolescents in psychological distress during hospitalization in a Psychiatric Inpatient Unit (*UIP*), aiming to understand their experiences and identify their challenges^(5)^. It is known that family full stay during hospitalization is required, and demands actions focused on their inclusion and follow-up, as the reorganization of aspects of their lifeworld directly impacts their physical and mental health, as well as their social and financial dynamics^(5–9)^. In view of the above, the study aimed to understand the experience of families of children and adolescents in psychological distress when faced with hospitalization in a *UIP* of a general university hospital.

## METHOD

### Design of Study

This is a qualitative, phenomenological-social study using Alfred Schutz’s theoretical-methodological framework, aimed at unveiling the phenomenon through social action, understood as an intentional act that has motivations projected by the individuals in their lifeworld, permeated by intersubjective, face-to-face relationships and their biographical situation, constituting their body of knowledge^(10)^. Moreover, the recommendations of the *Consolidated Criteria for Reporting Qualitative research (COREQ)* ^(11)^ were followed.

### Local

The study was carried out in a *UIP* of a university hospital in an inland city of São Paulo, which has sixteen mixed gender beds, with an average of four to five children or adolescents hospitalized per year. The multidisciplinary team consists of nurses, nursing technicians, psychiatrists, social workers, multidisciplinary mental health residents, and students from professional development programs. This unit was chosen because it is a field of practical activity and extension actions of the researchers involved in the study, and its initial approach occurred through the family care strategy adopted at the *UIP* through the weekly gathering of a group that embraces family members. It is one of the largest university hospitals in the country and a national reference center for tertiary services, focusing on research, teaching, service provision, and health actions aimed at the needs of patients treated in outpatient clinics and specialized inpatient units.

### Population

Family members who accompanied children and adolescents in psychological distress during their hospitalization at the *UIP* participated in this study. Furthermore, data collection also took place at the psychiatry outpatient clinic located in the same institution, on Wednesdays afternoons and Thursdays mornings, dedicated to the public aged 5 to 18, with an average of 80 to 100 services per month. In both services, the supervising nurse indicated children and adolescents who had already been hospitalized, as well as those who were hospitalized at the time of data collection. To access participants, the researcher went to the location and checked for the presence of family members who met the following selection criteria: being a family member of the child and/or adolescent in psychological distress who experienced hospitalization in the *UIP* at any time; and being over 18 years old. Family members who accompanied the child or adolescent at the outpatient appointment, but were not present during their hospitalization, were excluded, as were family members who did not attend scheduled appointments.

### Data Collection

Data collection was carried out in February and March 2022, using the phenomenological interview, which allows the subject who experiences the phenomenon to explain to the interviewer the meaning of the action developed in their world of intersubjective relationships^(10)^. The guiding question was: “What was your experience like during your family member’s hospitalization at the *UIP*?”, and from it, other questions were created that allowed the interviewee to delve deeper into the topic addressed. The interviews took place in a private location on the hospital premises, conducted by the researcher, recorded in digital audio, lasting an average of twenty-two minutes, with no refusal by the participants. It is important to highlight that there was no criterion related to the time elapsed between hospitalization and the interview, given that the phenomenon experienced is understood in the singularity of the family member’s experience, regardless of its temporality^(10)^. Theoretical saturation was reached based on the moment in which data repetition was identified, and no new elements, such as nuances, dimensions and variability, emerged to increase the revealed phenomena described in the categories^(11,12)^. The recommendation of a recent systematic review was followed with regard to confirming saturation from at least three consecutive interviews^(11,12)^.

### Data Analysis and Treatment

Based on the theoretical framework of social phenomenology, data were analyzed and categorized by the main researcher and the other authors who contributed to the study without using software. The steps indicated in studies based on the social phenomenology of Alfred Schutz were followed, in line with this research, including: full transcription of the participants’ statements; careful reading and re-reading of interviews to understand the meaning of family members’ experiences; and investigation of excerpts encompassing common meanings of the participants’ experiences and organization of speeches into thematic categories that express the “reasons why” and “reasons for” of what was experienced by the interviewees^(13)^. Social phenomenology and scientific literature on the topic addressed were used to carry out a comprehensive analysis and discussion of the results presented in the categories^(13)^. All interviews carried out were analyzed.

### Ethical Aspects

The study met all the criteria of Resolution No. 466/12, which regulates research involving human beings, and was approved by the Research Ethics Committee of the Universidade Estadual de Campinas under opinion No. 5.118.938 of 2021. The research participants signed the Free and Informed Consent Form before the interviews began. To guarantee anonymity to the participants, the statements were identified by the letter E as a way of representing the word “interviewee”, followed by the number corresponding to the chronological order in which the interviews took place.

## RESULTS

The study consisted of eight participants, five mothers, two fathers, and one grandfather, aged between 31 and 65 years old, all residents of the Metropolitan Region of Campinas, with a monthly salary income of one to four minimum wages, with only a farmer and a seamstress remaining working, since the others gave up their jobs to dedicate themselves to caring for children and adolescents in psychological distress. The interviews allowed the construction of the “we” relationship, and consequently to understand and organize the other’s experiences into two categories: the first expressed the participants’ stock of knowledge and bibliographic collection through the “*reasons why”* ^(10)^ and the second translated the expectations and objectives of the “*reasons for”* ^(10)^. The *“reasons why”* and the *“reasons for”* are described in the respective categories: *Family experience of hospitalization* and *Expectations of family members regarding the experience lived.*

### Family Experience of Hospitalization

In a scenario of hospitalization of children and adolescents in psychological distress, the family members participating in the study described the experience as an impactful moment, which led to changes in the family and the child’s routine, triggering the need to undergo treatment and start using medications for depression. Furthermore, the mother stopped working as a result of her son’s psychiatric condition.

*(...) the routine changed a little, his routine changed too. (E8)*

*I felt bad (…) last year, I went to a consultation, started taking medication, undergoing treatment... I had, like, depression (…) it was quite shocking. (E4)*

*And I’m a seamstress, but unfortunately due to (…) getting sick, I can’t work anymore. (E3)*

The family members’ experience of hospitalization also caused difficulties in the marital relationship due to the parents’ lack of knowledge on how to deal with their daughter’s situation. Furthermore, the mother demanded more from herself because she was a mother, and less from the father because she believed he did not know how to handle this type of situation; however, she recognized his attempt to understand and deal with the situation based on conversation. Before hospitalization, family members dealt with psychological suffering alone and, when they were unable to manage it, they felt like they were failing and wondering how to care.

*(…) I had a very difficult relationship with the father, also because I didn’t know how to deal with the situation. (…) And this of being mother, we end up demanding a lot from ourselves, not so much from the father, because the father doesn’t know how to deal with these things, at the same time they try, with a lot of conversation, to also understand how to deal with the situation, things fall into place. (E5)*

*Because we were carrying everything alone (…) And when we couldn’t handle it, we felt like we were failing, you know? How can we not take care of the girl? (…) I think that in reality we are carrying the weight alone (…) that was a lot of the weight I was carrying. (…) (E4)*

At the same time, the interviewees’ experience was marked by contrasting emotions: while it was good and comfortable due to the support the hospital provided, it was also bad and desperate because they were in a new environment. Participants found it difficult and strange to express their feelings, reporting that they themselves had difficulty understanding and explaining the sensation they experienced.

*I went through so much emotion. (…) Many ups and downs. (…) It’s a contrast, isn’t it?! Wow, there’s a bad side, but at the same time it’s good, because it’s part of it. (…) It was a while... It’s hard to say it was good. We don’t even understand each other (E7)*

*(…) at the same time we got desperate for being like this, in an environment that we wouldn’t want to be in, because it was completely new, but at the same time I felt comfortable with the support that the hospital gave me. (…) But it’s quite weird... It’s a feeling that you can’t explain, you just have to experience it. (E5)*

Family members also point out other difficulties, such as aggressiveness, the delay in the improvement of the acute condition, the first hospitalization, both for the child and the parents; the illness that affected the daughter and the family’s adaptation to the child who is no longer perfect as a result of psychological suffering. Concomitantly, the situation experienced gave rise to the feeling of pain, seen as natural and collaborative, as it is part of the life experience.

*But this time, we had to hospitalize her, she became very aggressive when she had this type of crisis... it’s very difficult. It takes a while to get back, it takes a long time... (E4)*

*It was very difficult, because I had never been hospitalized (…) She gave lots of trouble. (…) It’s difficult because seeing her ill, you know? Seeing her ill like that. I had never seen a person like that, I got to see her exactly her. (E2)*

*It was very difficult for us as a family, because she was a perfect child, you know? (…) It was a very difficult period of adaptation at the beginning (E5)*

*It’s a situation of great pain (…) But I said it this way: it’s interesting that with all the pain you still feel like “wow, I had this life experience”... Even so, she collaborates, she is part of... Pain is part of it (E7)*

During hospitalization, participants reported experiences of exhaustion and fear, as family members are worried about this being the child’s first hospitalization, and the fact that it is not a simple patient being admitted, but rather their daughter, and being concerned about the exposure to risks. The feeling of fear is also experienced when having to ensure that the child remains in the room during hospitalization, as the physical structure has characteristics that one is not used to, such as the bars, for example.

*So we were really exhausted like that, you know?! (…) We see that this way, it seems like a risk, you know?! I mean, so there could be abuse here, you could be surprised by a situation... The person who arrived with trauma may be more traumatized. (E7)*

*Fear, because he had never been hospitalized, and I know that if we were hospitalized we would be in a room, and the fear of what it would be like, because he (…) is an active teenager who doesn’t stop. (…) And so my fear was being able to keep him, right? (E8)*

*So, when I was approached and they said that she was going to be hospitalized, there was a very similar feeling, of fear, you know?! (…) So, when I came across the ward I saw that there were bars and everything, that it was a different ward from what I was used to (…) I was scared to know that it wasn’t just a patient, it was my daughter that would be going in there (E5)*

On the other hand, regarding hospitalization care, family members revealed that the team is helpful in the care provided, welcoming with affection, without aggression, and without ignoring us, in addition to being competent, professional, humane, with attentive doctors, talking and trying to find out what is happening. Families reported being treated well, as professionals pay attention to family members as well.

*The team is very good (…) in terms of service, everything was very good (…) I like the people here. (…) But I found them very competent, very professional staff. (E4)*

*I don’t know if the first world has a team like that, human, you know? (…) because they are attentive, talk, try to find out what is happening. (E6)*

*And the doctors are good, they treat with attention, (…) they sit and talk, (…) the nurses are also to be congratulated (…) Attitude is when the person arrives and pays attention to my son, then they arrive and treat him with affection, they don’t attack, they don’t say any words that might seem like the person wants to ignore that patient, you know? Here I feel that they do not ignore us. So I am treated well. (E1)*

*The service here was good, they treated us really well, you know? It helped a lot, so I liked it. (E2)*

The experience was marked by the support of the different professionals who make up the team, providing gymnastics, moments of conversation, to let off steam, and spaces to ask questions about the world of psychiatry. Treatment was seen by those interviewed as a set of situations beyond medications, carried out based on respect, through therapies and conversations with doctors and psychologists.

*The girls, both from psychiatry, psychology and social work, accompanied us the entire time and gave us great support. (…) The interns who proposed gymnastics for us, moments of conversation, even to clarify doubts about this universe of psychiatry. (E5)*

*There was also a psychologist, who was there every day to see how we were doing, if we needed anything, if I needed to let the steam off. (E8)*

*It was the treatment, the medications, the therapies, which he talked about with the psychologist, the things that the doctors talked about with him, and the medicines that the doctors gave him that solved the problem. (E3)*

In the family’s experience, seeing other patients with similar conditions being cared for and managed by professionals in the hospitalization space allowed for a satisfactory experience and the possibility of identifying this as the place to turn to in certain situations. At the same time, the family expressed a feeling of happiness for seeing treatment progress due to the competence of the hospital professionals and the participation in everything that was happening, feeling grateful.

*As she had never been hospitalized before, the first time was good, in the sense that I saw that there were people who knew, you know? (…) I think that for me it was good, for her it must have been horrible, but for me, in this sense of knowing that “there are other people with the same conditions, there are people who know how to care, who know how to manage, they have the place for me to go in these situations, if she gets like that. (E4)*

*I’m happy for him (…) What I’m feeling is that when I got here the doctors said he was very ill, now I’ can see a lot of progress (…). (E1)*

*I was happy because I knew that my son was in a hospital that had competent doctors to take care of him. And I was happy that they accepted me, that I was there participating in everything that was happening (…). (E3)*

### Expectations of Family Members with the Experience Lived

The expectation of family members interviewed in relation to hospitalized children and adolescents in psychological distress is that they leave the hospital recovered, as the team, together with the medicine, is trying to do their best. Furthermore, they hope that the family member will be a person like everyone else, and will recover their mental health so they can return to work.

*I hope he leaves here recovered, the doctors and staff are doing everything they can, you know? (…) I hope my son is a person like you one day in his life, you know. (…) Waiting for him to sleep and for the medicine to give him a little more help, to slowly recover his mental health, so that he can, one day, go back to work as before... he worked in the garden, he sold his vegetables (…) (E1)*

Concomitantly with this recovery, those interviewed are concerned about the lack of autonomy of children and adolescents in relation to psychological suffering, questioning themselves about who will stay and care for them, since before they had hope that the condition would not return; however, they now understand that it could worsen again.

*And the concern about her not being autonomous, you know? We question: who will be with her, who will take care of her? Because she doesn’t have any autonomy, so that’s what worries me a lot (...) because before we were... we had a little hope that “no, it won’t happen again”, not today, today I already understand that it can happen again. (E4)*

With their experience during hospitalization, the study participants wanted the team to have basic preparation to deal with the family member’s health status, recognizing paths for treatment that would not worsen the condition. Furthermore, the family expected the team to seek professional growth, so that they could, this way, work better and provide good treatment, in addition to the medication supply, to their patients, contributing to their progression.

*(…) they should have at least the basics of a person in a state like this. There are ways for treatment, because that can get worse (…) They seek this growth that is good for them (…) working better, I think patients will be better treated, it will even improve more, it will collaborate with the treatment. So it would be the medicine and a good treatment (…) (E7)*

Family members expect a change in the physical structure of the unit, with the division of space between age groups and biological sex, because, the way it is currently organized, everyone stays together, and they hope that with the restructuring it will be possible to carry out some type of activity other than lying in bed.

*(…) It’s really a question of space, but I think there should be a greater division between age groups, because everyone ends up crowded together, you know? Even to have some type of activity (…) besides lying in bed.* (E4)

*There could be a separation of space, although I know there’s a lot involved, but (…) they had to have this orientation, have this view like “wow, let’s avoid it, let’s find how we can separate it, that here they’re under 18, here is a for women”.* (E7)

## DISCUSSION

The results of this study indicate that the families of children and adolescents hospitalized in psychological distress consider the experience of hospitalization as an impactful moment, which led to changes in the family routine and difficulties in the marital relationship. The impact experienced may be due to the change in environment, as the hospital is a different place, with multiple equipment and unknown individuals, in addition to being a source of different emotions, as shown in a study carried out with family members accompanying hospitalized children^(14)^.

These repercussions can be interpreted from Schutz’s phenomenological perspective, in which it is understood that the hospital, although not part of the family’s lifeworld, may have records of family hospitalization experiences^(10)^. Thus, its knowledge base can offer adaptive resources to its natural attitude, so that it can live in this scenario with many new features, such as the routines of a *UIP*, team dynamics, or even hospital equipment^(10)^.

There is a need to restructure the family’s routine, as the accompanying individual no longer plays their role as before in the family nucleus to be with the hospitalized child and adolescent and be able to meet their demands and adapt to the new reality imposed^(7,15)^. The literature shows that this change can be considered by family members as indifferent or positive, as it unites the family in difficult situations of crisis^(7,8)^. Moreover, as it is correlated with the disconnection from paid activities, lack of freedom, and contraction of social life, it can, consequently, lead to the isolation of those who care, as well as the abandonment of self-care and feelings such as sadness^(7,8)^.

Sadness was an experience expressed by the family members interviewed, progressing to depression, requiring drug treatment. However, there are authors who state that caregivers of individuals in psychological distress, due to the burden of care, have a predisposition to illness, and in a study in a *UIP*, family members also pointed out depression as a result of the episode experienced^(8,16)^.

The findings of this research showed the interruption of work by the family due to the child’s psychiatric symptoms, evidence in line with the literature, as previously pointed out, and in accordance with a cross-sectional study, which analyzed that 85.9% of caregivers of children and adolescents in psychological distress gave up paid work to dedicate themselves to caring for them^(17)^.

The aforementioned changes, especially the need to reorganize the routine, can weaken the emotional bonds among family members and compromise the marital relationship^(15)^. A survey of caregivers of children with mental disorders showed that 81.3% of participants claimed changes for the worse in their marital life^(17)^, data that corroborates the results of this research. In a study carried out in London, on the perception of paternity of children with intellectual disabilities, some parents indicated the opposite, claiming that marriage was strengthened as they had to work together and be closer to each other to better cope with their child’s illness^(18)^.

In the family scenario, participants reported that the mother demands more from herself because she is a mother, and less from the father because of the belief that he does not know how to manage his daughter’s symptoms, despite recognizing his attempt to deal with the situation through conversation. It is known that, even in contemporary times, gender stereotypes permeate family relationships and influence the functioning of fatherhood and motherhood, as they stipulate the moral and cultural values of the family nucleus and determine the appropriate and ideal roles of men and women^(19)^. As a consequence, there is the naturalization of the mother’s figure as a caregiver and provider of affection, and the father’s figure as a financial provider^(19)^.

The father’s involvement in children’s care is observed only as complementary to that of the mother; therefore, it is essential that the healthcare team contributes to the denaturalization of the maternal figure as the main and only person responsible for the child’s care^(19)^. The inclusion of the father and other members of the user’s affective network in their *PTS* calls on them to actively participate in care, education, practices, and the like required for children and adolescents’ healthy development^(19)^.

Another important finding demonstrates that, before hospitalization, family members were unable to manage psychiatric symptoms, felt like they were failing and questioned themselves about how to provide care. Based on Schutz’s phenomenological perspective, one’s natural attitude has to undergo some adaptation to experience something new, with psychological suffering also being seen as a new phenomenon to be unveiled and included in the family’s knowledge base^(10)^. At the same time, research reveals that the family reports not feeling prepared to deal with the situation of having a member with a mental disorder and expresses anxiety, embarrassment, and impotence for not knowing how to act in situations of crisis, and accepts their hospitalization^(8,15)^.

The changes in the family’s lifeworld, combined with the stay in the *UIP* and the experience of psychological suffering, can make family members vulnerable, triggering the experience of tiredness, stress, anxiety and physical, emotional and psychological exhaustion^(15)^, besides making ambiguous feelings such as fear, sadness, anguish, shame and guilt emerge, which represent negative experiences, and zeal, affection and gratitude, which express positive feelings^(8,15)^.

This contrast of emotions was also reported by the participants in this study, who described the experience as good, comfortable, bad, and desperate, in addition to experiencing exhaustion. The experience of hospitalization gave rise to the feeling of pain, seen as natural as it is part of the life experience. In a survey of family caregivers of people in psychological distress, pain was also evidenced due to the act of caring, with the need for the family to assign meanings to this action and this suffering, such as mission, God’s will, love, obligation, among others^(8)^.

The need to include families in care in the hospital context is evidenced through collective spaces, such as family reception groups and individual approaches. It is necessary to promote training and interventions with the health team to raise awareness about the importance of specialized embracement, management of relationships, adequate communication and forms of care in what regards the family^(20)^. Their inclusion in care allows the construction of bonds and the sharing of experiences, helping family members have a more pleasant experience, qualifying care^(20)^.

The subjects highlight the difficulty and strangeness they have in expressing their feelings arising from the hospitalization experience. It is understood that family members interpret the experience lived together through their knowledge base and their biographical situation^(10)^. Thus, when they witness a new phenomenon, in this case, the experience of hospitalization of children and adolescents in psychological distress, they may have difficulty expressing what they feel, since they still do not have sufficient repertoire in their knowledge base to interpret it.

In addition to the expression of feelings, the subjects report other difficulties experienced, such as the delay in improving the acute condition and the aggressiveness that the child and adolescent may be showing. Symptoms such as aggressiveness, agitation, and delirium require greater effort to stabilize and can more easily stimulate crises, with aggressiveness being highlighted by families as an element that hinders family relationships and coexistence, and can cause fear, insecurity and anguish^(16)^. Anxiety also appears when there is an exacerbation of psychological suffering, associated with sadness, frustration, impotence, and an increase in subjective overload, as crises lead families to believe that they are incapable of adequately caring for and providing the necessary support for the child and adolescent^(7,21)^.

In moments of crisis or exacerbation, family members identify the hospital as the place they should go, corroborating the findings of this study^(21)^. Nevertheless, the authors of this study understand that the hospital must be identified as a locus of transitional care, as the results effectively demonstrate the need to expand and make the embracement of family demands more flexible, promote bonding and trust, as well as to emphasize care and longitudinal follow-up in *RAPS* territorial services. This means that caring for family members and caregivers is not always talking and intervening in relation to the demands of children and adolescents, but rather considering the processes that parents and caregivers themselves go through, mainly related to feelings of fear, guilt, chasing and, at times, emotional overload^(9)^.

Fear was a feeling experienced by family members and deserves to be highlighted in this discussion, as it can originate from the perception of the disease that affects the children, which involves the parents’ adaptation process to the child who is no longer perfect. Studies carried out indicate that the family undergoes different impacts due to psychological suffering, believing that their family member is now a compromised and limited person^(8)^.

After becoming aware of diagnostic hypotheses of psychological suffering, family members showed varied reactions, “such as acceptance, concern, suffering, denial, a feeling of impotence, and anticipation of turbulent moments”^(22)^. This experience raises the important discussion of the support required for the longitudinal care of families and children and adolescents in psychological distress, and the authors of this manuscript reinforce the perspective of promoting psychosocial rehabilitation actions to strengthen belonging and reduce stigma.

Another piece of evidence from this study deals with family members’ expectations that children and adolescents leave the hospital recovered, and that they become people like everyone else, so that they can move around the territory, work and, in addition, rescue their autonomy through therapeutic actions in the follow-up and mental health care. Recovery is a very common ambition among family members, who hope for a state of normality, with remission of psychiatric symptoms, so that their family member can live actively and independently, and the expectation of recovery supports the family during coping of this process adversities^(7,16)^.

To achieve autonomy and strengthen the social life of children and adolescents in psychological distress, the creation of means that enable the expansion of these individuals’ potential is required, which can occur through co-responsibility for care in a territory within the family, society and work of a multidisciplinary team, which establishes planned interventions and provides support so that the relationship among the different actors takes place with no judgment^(16)^.

Considering the reasons, the division of space between age groups and biological sex was also pointed out by family members as an expectation, as they believed that, this way, it would be possible to carry out activities other than lying in bed. A study carried out with nurses about their perception of the mental health environment indicated the precariousness of the service infrastructure, generating inadequate assistance due to the constant improvisation to which they are subjected, and revealing the need to readjust the environments, considering the particularities of mental health care, for the development of effective therapeutic interventions^(23)^. The family also recognized the physical space as being decisive and contributing to the patient’s treatment, with hope that it provides opportunities for interpersonal relationships that are beneficial to the subject^(24)^.

Regarding the physical space and structure, the participating family members reported their concern about the risk of abuse occurring within the ward, bringing to light the experience of fear due to the uncertainty of ensuring that their child would remain in the *UIP* during the time of hospitalization. A study shows that, in a *UIP*, staff and family members are constantly concerned about the possibility of sexual relations between patients during the hospitalization period, and add this risk to the issues of the physical environment, such as the presence of subjects of both sexes in the same space, without a separation between them^(23)^.

Furthermore, the physical structure was also identified as a factor generating fear in family members, as they are not used to the hospital environment and hospitalizations, all of which is intensified by a *UIP* with bars. The scenario where human beings live and build their social relationships is characterized in a unique way in the lifeworld, based on each subject’s own interests, motives, ideologies, and the like, in such a way that their previous and lifelong experiences guide them about the way of behaving and thinking in the social environment^(10)^. These experiences together constitute the individual’s knowledge base, which is accessible and used to interpret their past and present experiences^(10)^. It is understood that family members are surprised by new scenarios when experiencing hospitalization and, when faced with a different environment from which they have references, feelings, such as fear, are generated during their adaptation process.

The experience of family members during hospitalization was also positive due to the support provided by the hospital team, the perception of other patients with similar conditions, treatment progress, with the latter being understood as a set of situations that go beyond medications, carried out with respect and starting from therapeutic actions. Support from the healthcare team to family members provides them with learning to better care for children and adolescents^(25)^. The family’s close contact with the UIP provides the opportunity to clarify their doubts, acquire greater knowledge about the therapeutic method and the progression of treatment, and provide guidance, since this set of factors favors the improvement of care by the family, who feels encouraged to participate in the *PTS* ^(24,25)^.

The lack of specific knowledge on the part of the team was highlighted in a study, in which professionals from a pediatric ward care for mental health cases, but recognize that this care is hampered due to several limitations, such as the lack of satisfactory training, absence of specialists, inadequate physical structure, and unpreparedness to deal with the issue^(26)^. Therefore, there are gaps in the training of health professionals regarding knowledge about the mental health of children and young people, which hinder and limit their interventions in these cases, leading to fragile and unsatisfactory assistance^(26)^.

As an implication for practice, the importance of group or individual spaces that allow the family to be embraced, heard, have their doubts clarified, exchange experiences with other families to learn about their experiences, and establish bonds with the team who, based on qualified listening and humanized reception, can give voice to these family members^(24,26)^.

The action of coexistence with other family members who are experiencing a similar biographical situation of child hospitalization favors their bonding, due to mutual understanding, through which they share experiences and build relationships of assistance and coping, using their knowledge base. They function as a support network for family members, exposing the need that these families have to be heard and understood^(24,26)^.

## CONCLUSION

This study allowed knowing the experience of families of children and adolescents in psychological distress during hospitalization from the theoretical-methodological perspective of Alfred Schutz’s social phenomenology.

When experiencing the moment of hospitalization, family members began to restructure their lifeworld, from their routine to work and marital and emotional relationships. Furthermore, they experience contrasting emotions and ambiguous feelings, such as fear, sadness, anguish, shame and guilt, representing negative experiences, and zeal, affection and gratitude expressing positive feelings during hospitalization, with the hospital being recognized as a space where they must seek care.

The expectation that children and adolescents present conditions of mental normality permeates the participants in the moments after hospitalization, and it is possible to observe that longitudinal care for children and adolescents and their family network in specialized health services distributed in the territory is required.

As an implication for practice, the importance of building individual and collective care spaces for family members was highlighted, with the presence of a multidisciplinary team that, through the professional-family relationship, considers the different processes through which parents and caregivers themselves go, especially those related to the overload of emotions and expectations experienced regarding hospitalization, expanding and implementing care and embracement of family members’ demands. Further studies shall be carried out in other health services, and the performance of the study in a hospital unit can be understood as a limitation.
